# Supplementary material for: Single-Cell Profiling of the Developing Organ of Corti Identifies Etv4/5/1 as Key Regulators of Pillar Cell Identity
Source: bioRxiv. 2026 Jan 20:2026.01.19.700450. Preprint. [Version 1] doi: 10.64898/2026.01.19.700450 (PMC12892159; doi:10.64898/2026.01.19.700450)

Sakamoto and Kelley, Supplemental Figure 1

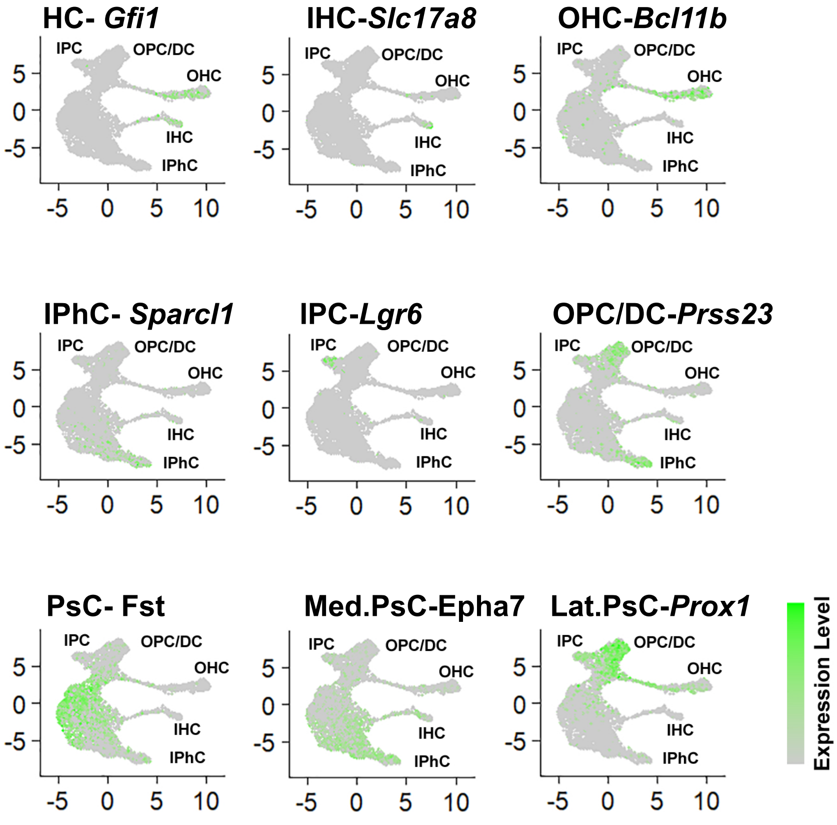

Sakamoto and Kelley, Supplemental Figure 2

PsC to Lat.PsC Transition

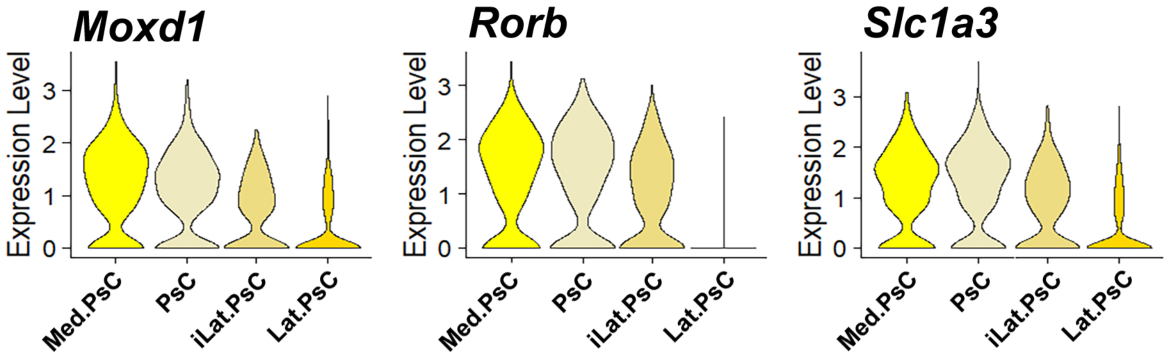

PsC to lPhC Transition

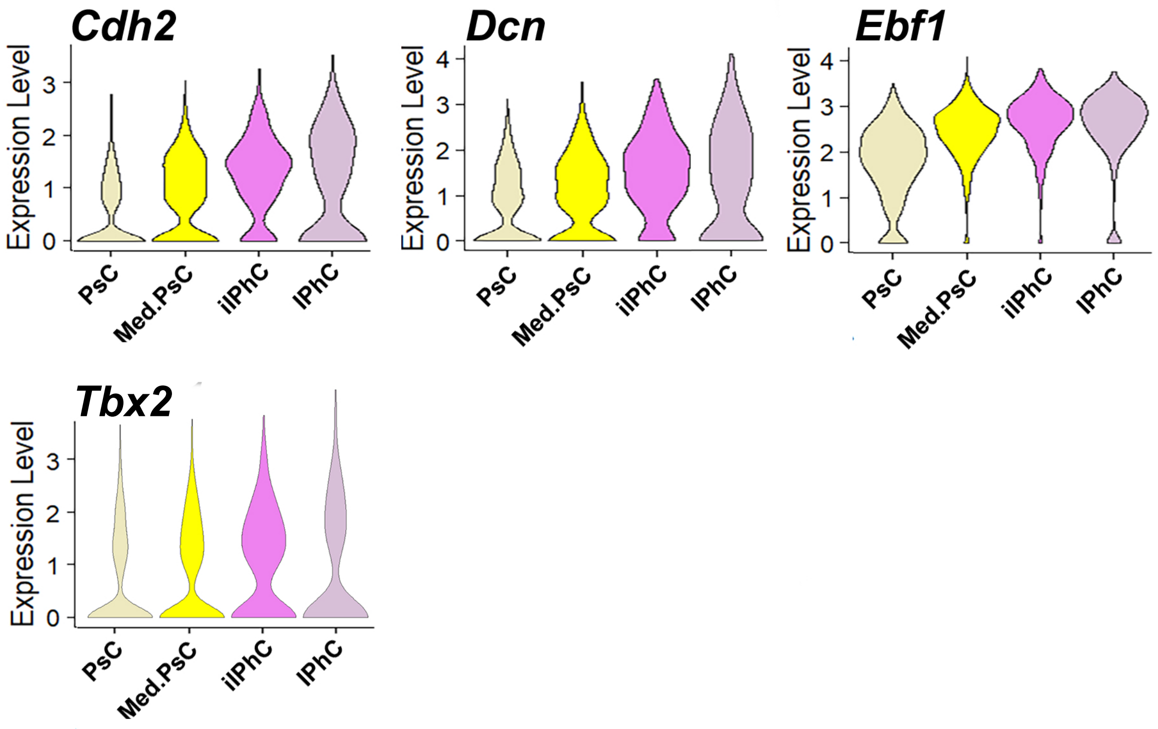

Sakamoto and Kelley, Supplemental Figure 3

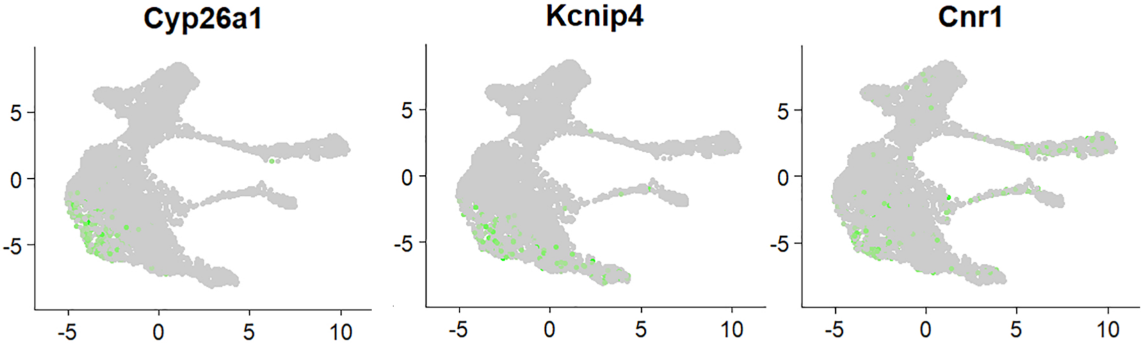

Sakamoto and Kelley, Supplemental Figure 4

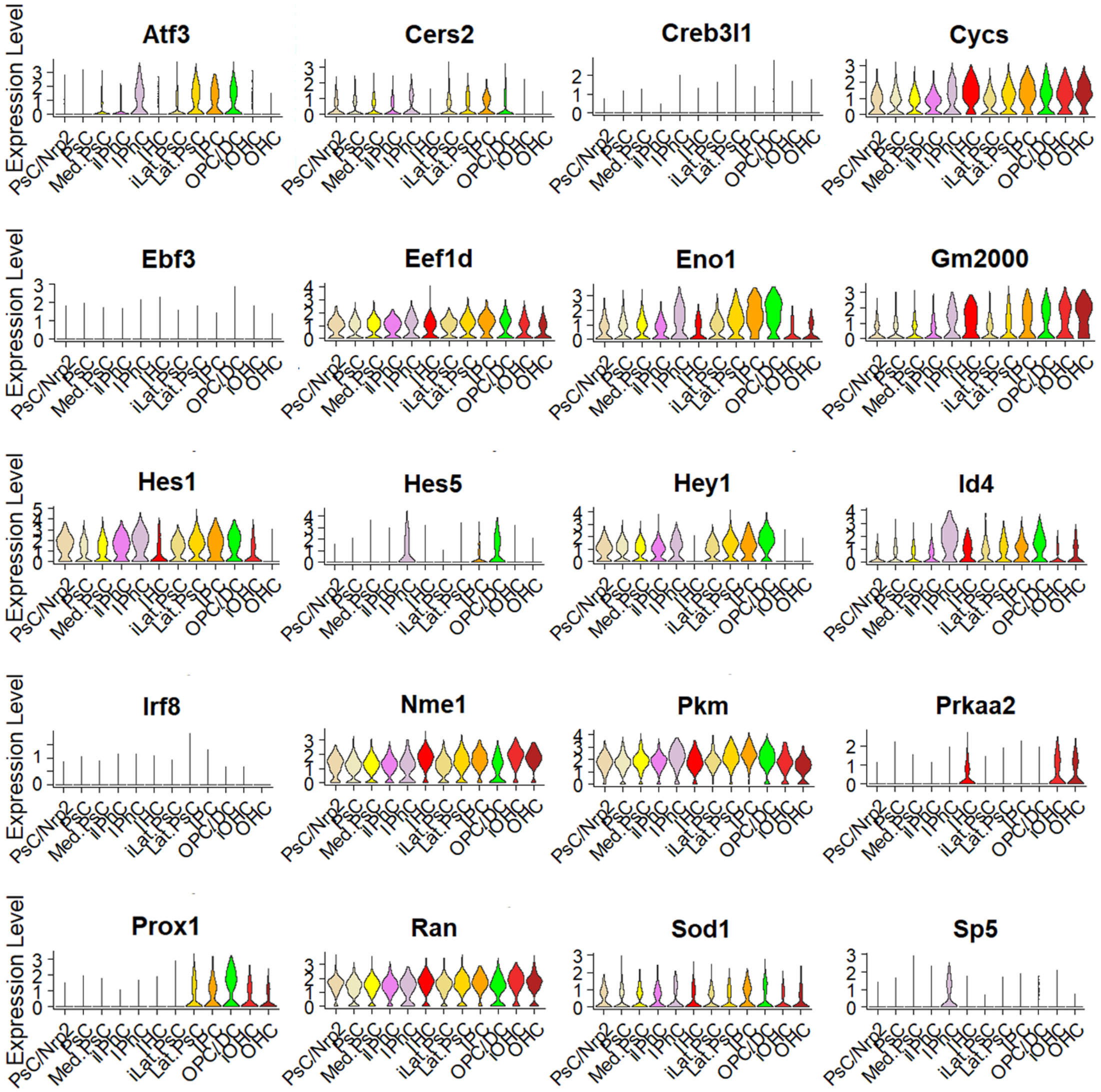

Sakamoto and Kelley, Supplemental Figure 5

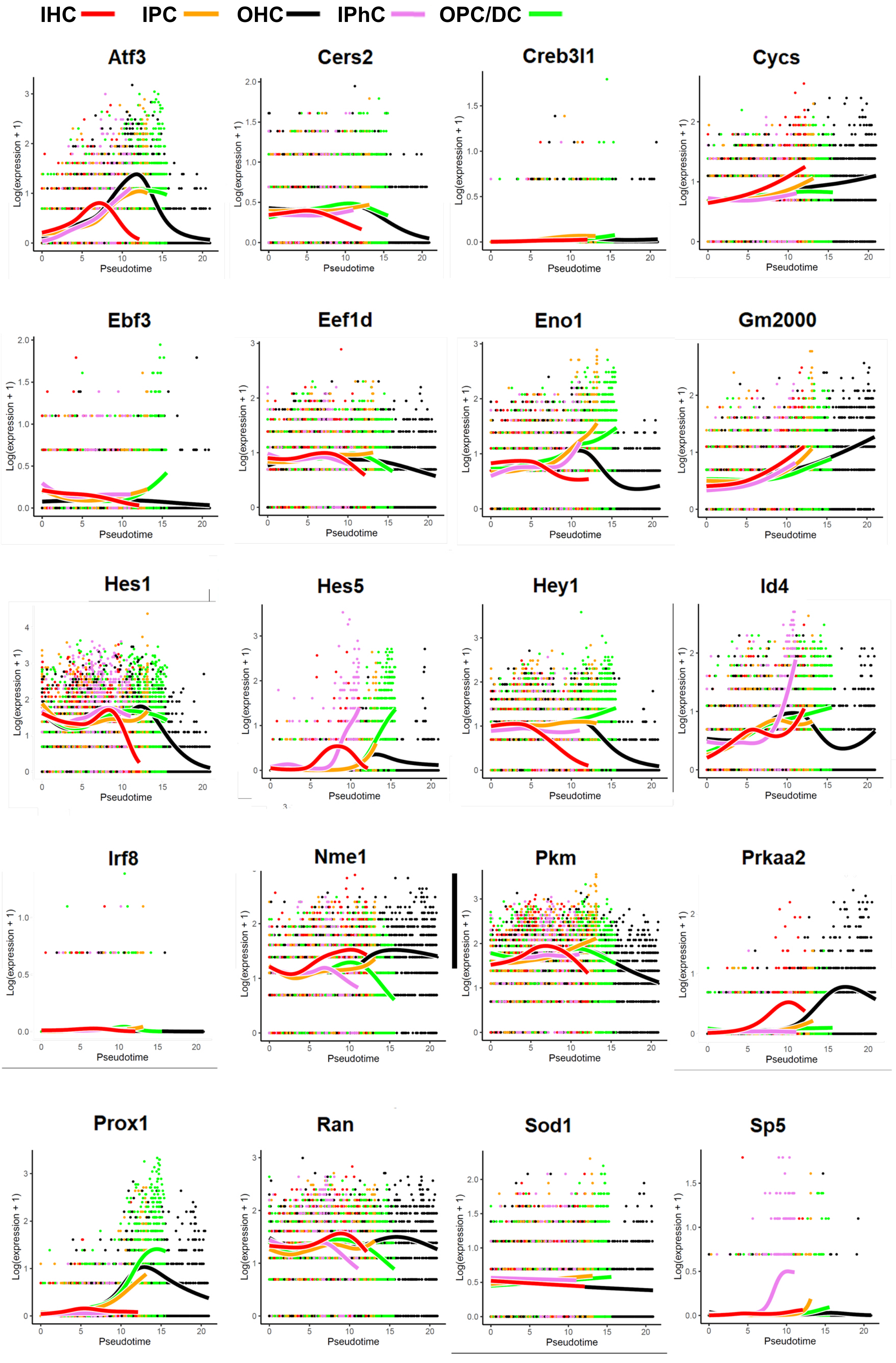

Sakamoto and Kelley, Supplemental Figure 6

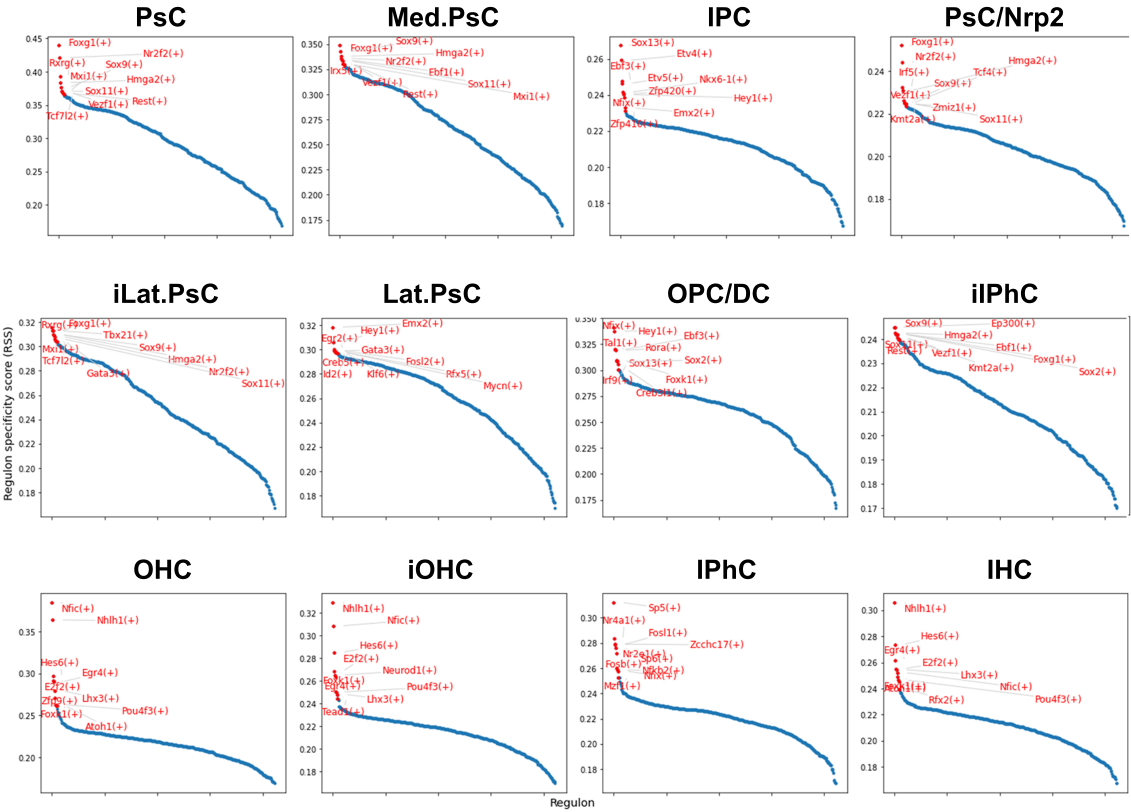

Sakamoto and Kelley, Suppl. Fig. 7

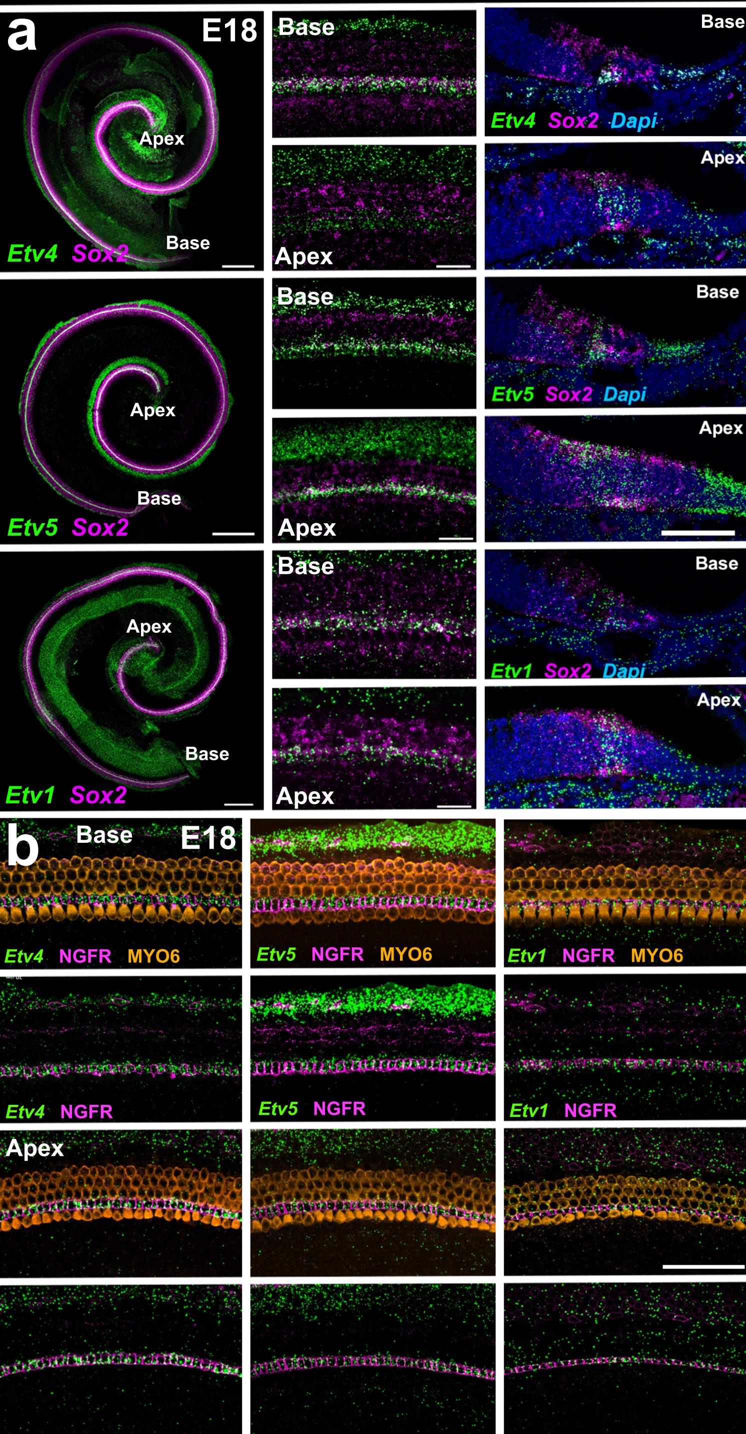

Sakamoto and Kelley, Supplemental Figure 8

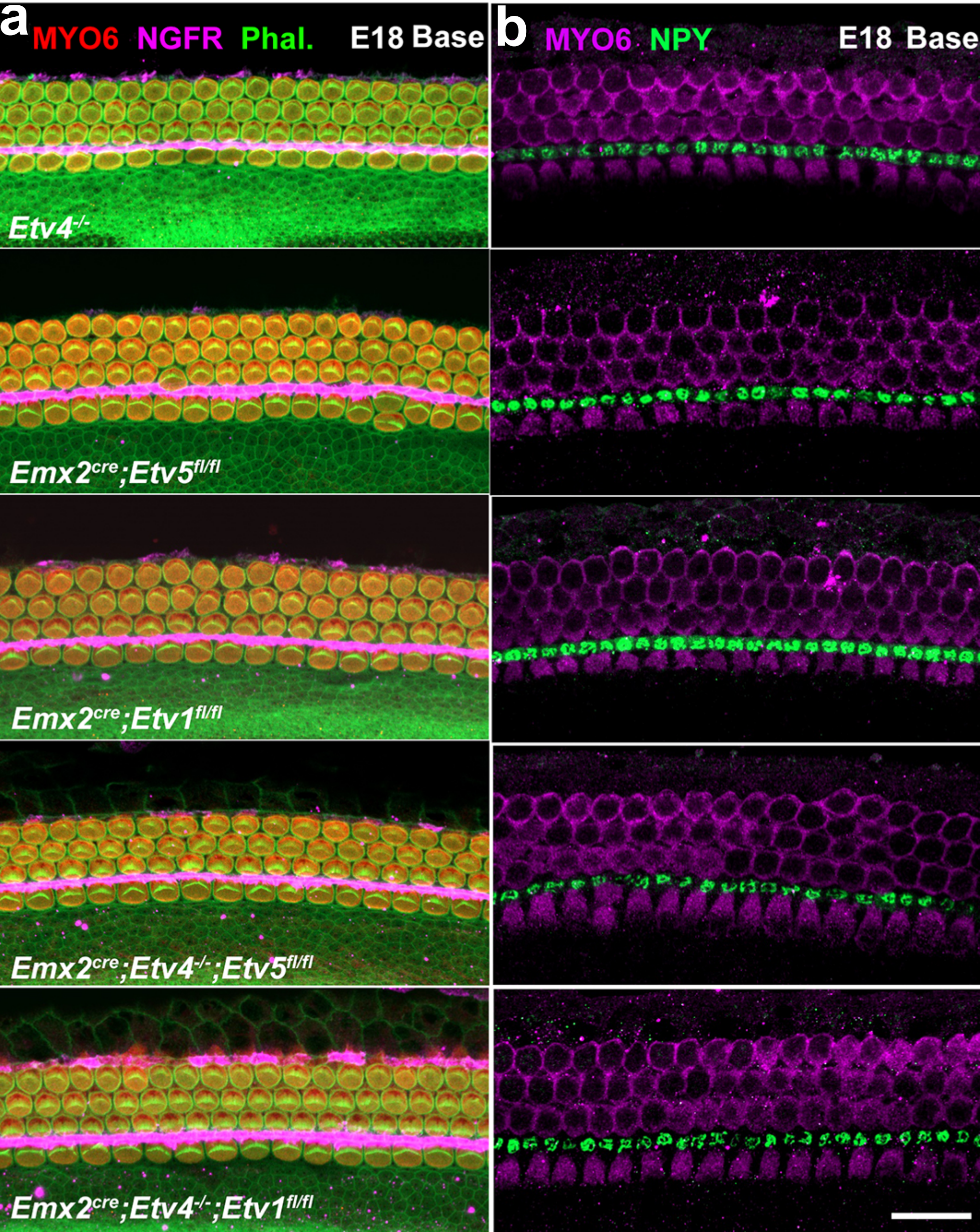

Sakamoto and Kelley, Supplemental Figure 9

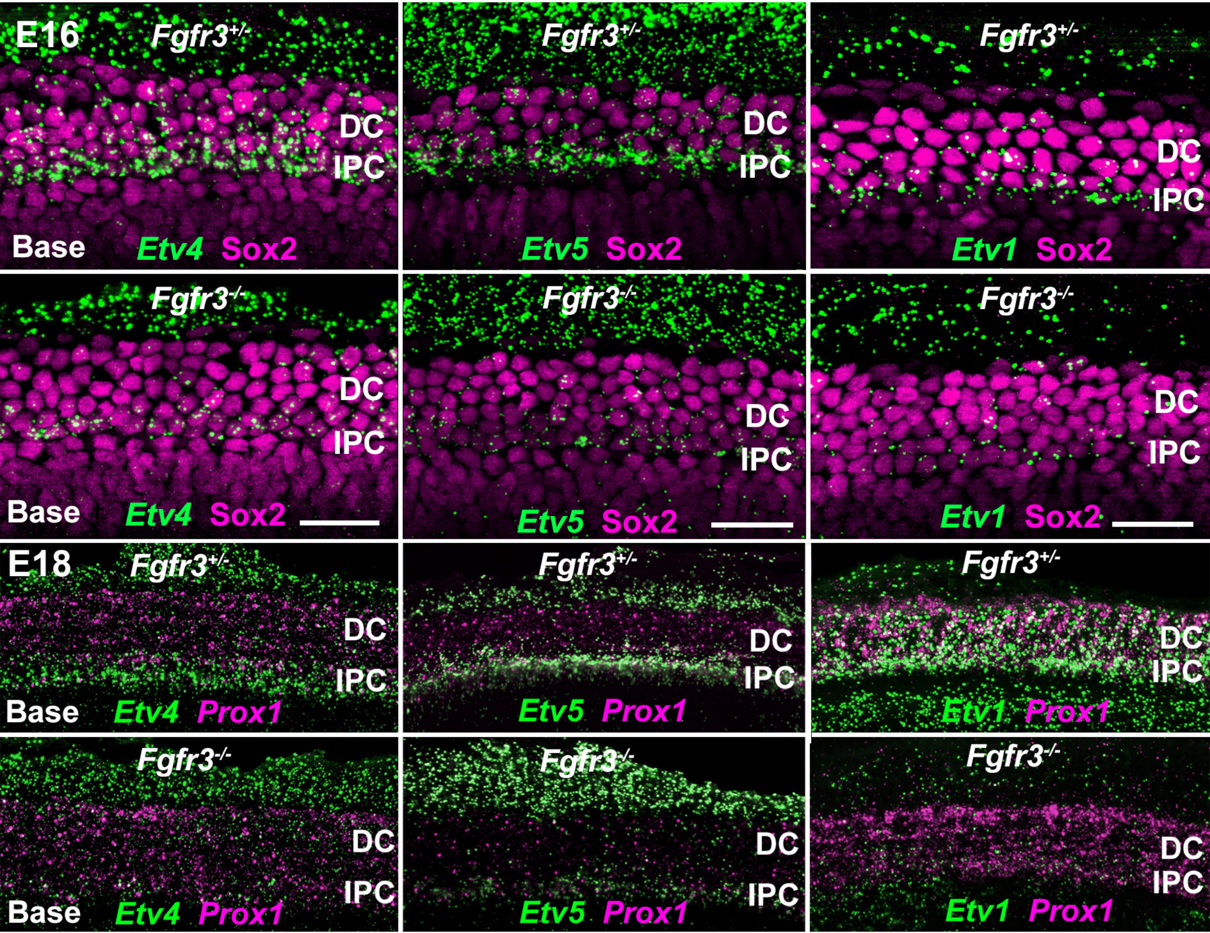

Sakamoto and Kelley, Supplemental Figure 10. Changes in *Etv* Target Gene Expression in *Fgfr3*$^{-/-}$ Cochleae

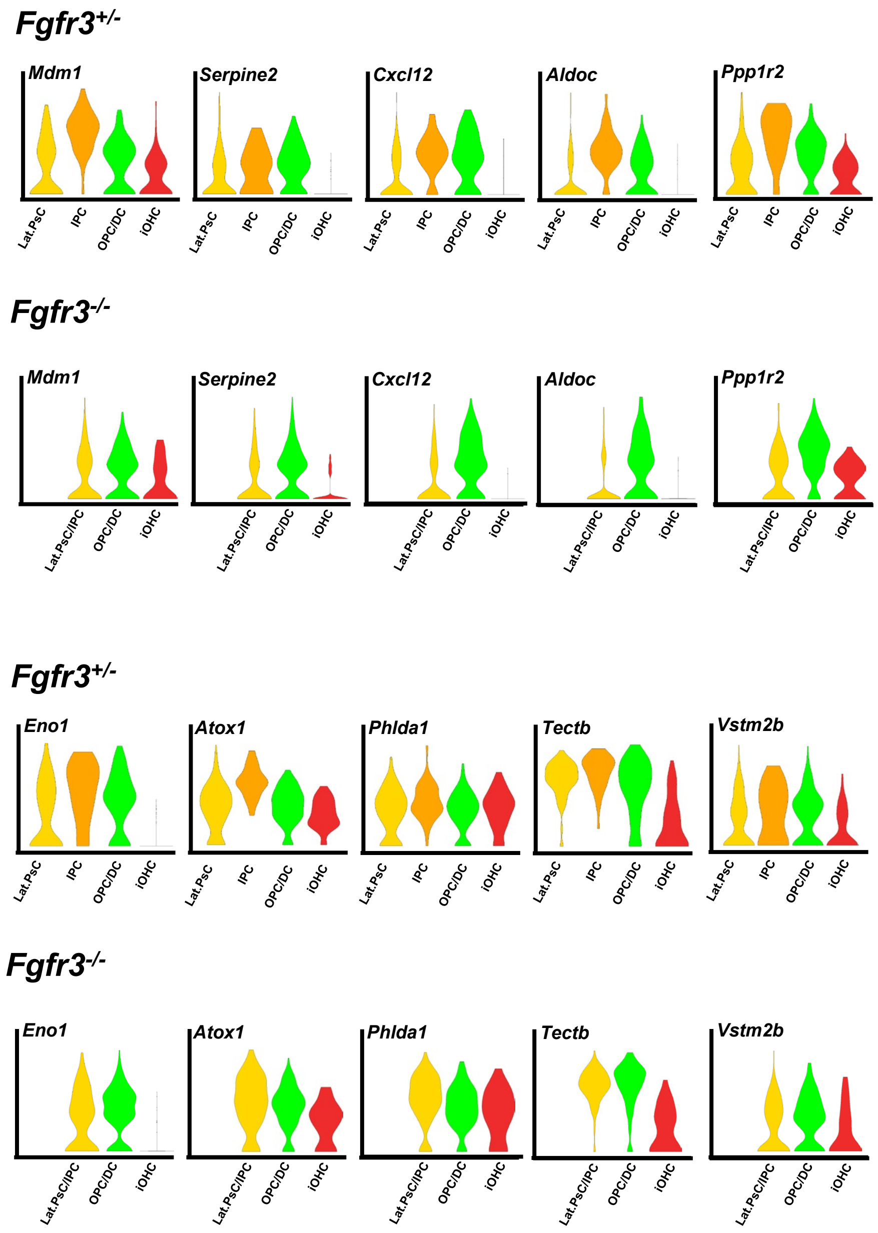

Sakamoto and Kelley, Supplemental Figure 11.
Changes in OHC and OPC/DC Gene Expression in *Fgfr3$^{-/-}$* Cochleae

OHC Genes

Fgfr3$^{+/-}$

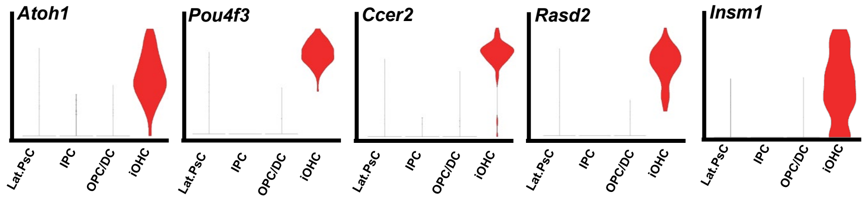

Fgfr3$^{-/-}$

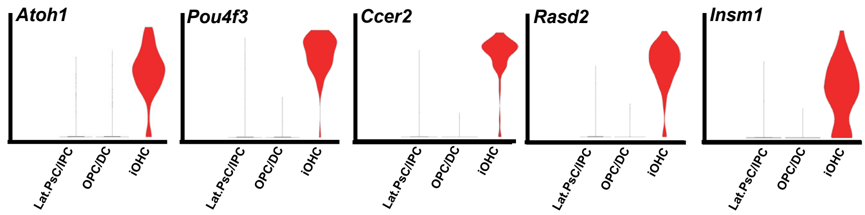

OPC/DC Genes

Fgfr3$^{+/-}$

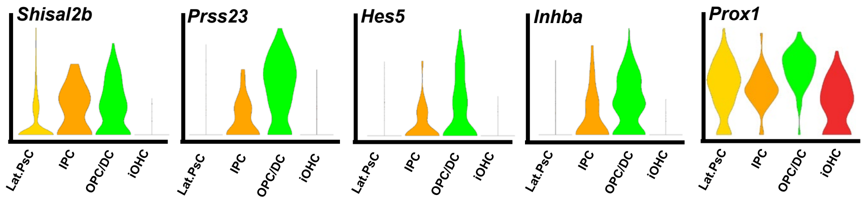

Fgfr3$^{-/-}$

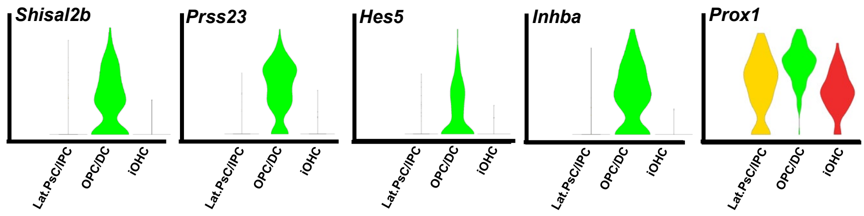

Supplement: Supplement 1 [file media-1.pdf]
